# Draft Genome Sequence of *Pseudomonas* sp. Strain ICMP 22404, Isolated from Solanum lycopersicum Plants with Pith Necrosis Symptoms

**DOI:** 10.1128/MRA.00649-19

**Published:** 2019-08-22

**Authors:** Luciano A. Rigano, Merje Toome-Heller, Katharina M. Hofer, Brett J. R. Alexander

**Affiliations:** a Plant Health and Environment Laboratory, Biosecurity New Zealand, Ministry for Primary Industries, Auckland, New Zealand; b Instituto de Biología Agrícola de Mendoza, CONICET-Universidad Nacional de Cuyo, Mendoza, Argentina; University of Arizona

## Abstract

We report here the draft genome sequence of Pseudomonas sp. strain ICMP 22404, isolated from Solanum lycopersicum plants showing pith necrosis symptoms. The draft genome size is 6,686,400 bp, consisting of 86 contigs with a G+C content of 60.7% and containing 5,876 coding sequences, 60 tRNAs, and 11 rRNAs.

## ANNOUNCEMENT

Pseudomonas is a diverse genus of Gammaproteobacteria whose members include known plant pathogens that infect economically relevant crops, including tomatoes (1). We isolated a Gram-negative aerobic bacterium from tomato plants showing pith necrosis symptoms in New Zealand. The isolation was made by plating the bacteria oozing from the symptomatic plant stem onto King’s B agar (KB) medium and incubating plates overnight at 25°C. One colony was picked and restreaked onto a new KB plate, and an isolated colony from this plate was used to make a stock from where cultures were grown for subsequent DNA purification and strain deposition in the International Collection of Microorganisms from Plants (ICMP). Initial PCR and Sanger sequencing of a portion of the 16S gene with primers RP1 and FD2 (2) confirmed that this strain belongs to the genus *Pseudomonas*; however, species-level identification proved to be problematic due to limitations that are inherent to this approach (3).

Total DNA for genome sequencing was extracted from a culture grown in nutrient broth (Difco, USA) overnight at 25°C with vigorous shaking. Cells were harvested by centrifugation, and DNA was extracted using a DNeasy blood and tissue DNA purification kit (Qiagen, USA) according to the manufacturer’s instructions. One microgram of DNA was randomly fragmented by sonication, and a library was constructed using a NEBNext Ultra DNA library prep kit for Illumina (New England BioLabs, UK) following the manufacturer’s instructions. The resulting library was sequenced on an Illumina HiSeq platform using a 2 × 150-bp paired-end configuration, generating a total of 22 million raw reads (Genewiz, China).

Low-quality read filtering and *de novo* assembly were performed using the Shovill pipeline (version 1.0.4; https://github.com/tseemann/shovill), which employs Trimmomatic version 0.38 (4) and SPAdes version 3.12.0 (5), using default settings. Contigs smaller than 200 bp were not considered. The draft genome size of the isolate is 6,686,400 bp, and it is distributed in 86 contigs with a G+C content of 60.7%. The draft genome assembly has an *N*_50_ value of 303,405 bp and an estimated mean coverage of 296×. Annotation of the draft genome sequence was performed using the NCBI Prokaryotic Genome Annotation Pipeline (6), and the final annotation was manually curated to ensure correct start/termination sites and gene identity. The annotation process identified 5,876 coding sequences, 60 tRNAs, and 11 rRNAs in the draft genome of this bacterium.

The genome sequence of *Pseudomonas* sp. strain ICMP 22404 will provide insights into the biology, mechanisms of virulence, and taxonomy of this microorganism.

### Data availability.

This whole-genome shotgun project has been deposited in DDBJ/ENA/GenBank under accession no. VCNG00000000, BioProject accession no. PRJNA544572, and BioSample accession no. SAMN11843475. The version described in this paper is the first version, VCNG01000000. Raw sequencing reads for this project can be found under SRA accession no. SRR9722508. *Pseudomonas* sp. strain ICMP 22404 was deposited in the International Collection of Microorganisms from Plants (New Zealand).
